# Outcomes of percutaneous ultrasound-guided A1 pulley fenestration release with small-gauge needles for treatment of trigger finger

**DOI:** 10.1007/s00256-025-05028-8

**Published:** 2025-09-06

**Authors:** Faysal F. Altahawi, Gregory Owendoff, Eugen Lungu, Michael Forney

**Affiliations:** 1 Department of Diagnostic Radiology, Cleveland Clinic, Cleveland, OH USA; 2 Cleveland Clinic Lerner College of Medicine, Cleveland, OH USA; 3 Department of Radiology, Centre Hospitalier de L’Université de Montréal, Montreal, QC Canada

**Keywords:** Trigger finger, A1 pulley release, Fenestration release

## Abstract

**Objective:**

To retrospectively evaluate outcomes of an ultrasound-guided A1 pulley fenestration release technique using small-gauge (hypodermic or spinal) needles for the treatment of trigger finger (TF).

**Materials and methods:**

A retrospective chart review of all TF fenestration release procedures performed by two musculoskeletal radiologists between July 2020 and August 2024 was conducted. The technique included a steroid injection after release. Preprocedural and postprocedural functional Quinnell grades and any immediate complications from the procedure report were primary outcome measures. Clinical pain score, functional outcomes, other TF interventions, and delayed complications were secondary outcome measures.

**Results:**

A total of 119 procedures were performed in 92 patients (61% women, mean age 63 ± 13 years), with 95 procedures (80%) following prior TF injection with refractory symptoms. Periprocedural Quinnell grades were reported in 99 procedures (83%), with immediately improved scores for all (median-preprocedural-to-postprocedural, 3-to-0; *p* < 0.001). Retrospective follow-up data were available for 60 procedures (50%), of which 70% experienced functional improvement. Pain scores were significantly improved at follow-up (median-preprocedural-to-follow-up, 4-to-0.5; *p* = 0.046). Subsequent interventions occurred following 22 cases (18%), of which 15 (13%) required additional steroid injections, 3 (3%) required repeat fenestration, and 5 (4%) required surgical release. No immediate or delayed complications were otherwise reported. Higher Quinnell grade at end of procedure predicted increased rates of follow-up (OR = 3.17, p = 0.012) and suggested worse functional status at follow-up (OR = 0.25, *p* = 0.054), and smaller peri-procedural improvement increased odds of additional intervention (OR = 0.48, p < 0.001).

**Conclusion:**

Ultrasound-guided fenestration is an effective and safe treatment for TF in the outpatient setting.

## Introduction

Stenosing flexor tenosynovitis, or trigger finger (TF), is an often debilitating pathology of the digital flexor tendon sheath with an estimated lifetime risk of 2% to 3% in the general population and up to 10% in diabetic individuals [1, 2]. This condition occurs most often in the fifth and sixth decades of life and is more common in women [3]. In addition to diabetes, other proposed risk factors include a history of frequent repetitive movements, traumatic injury, rheumatoid arthritis, and carpal tunnel syndrome [1, 4].

Patients with TF experience a mechanical dysfunction of the flexor tendon as it passes through the first annular (A1) pulley at the metacarpophalangeal joint. Reactive tendon hypertrophy and ligamentous narrowing of the A1 pulley cause a relative stenosis of the pulley as the tendon passes through it during finger movement, exacerbated by the natural increase in flexor tendon diameter during flexion [5, 6]. Clinically, this manifests as clicking, catching, stiffness, pain, or swelling proximal to the metacarpophalangeal joint, often requiring passive manipulation to extend the affected digit [1, 5]. This condition is usually diagnosed clinically; however, ultrasonography is playing an increasingly important role in characterizing the affected anatomy, assessing severity, and guiding intervention [7].

Treatment of TF aims to restore tendon function and alleviate pain. Initial management includes the use of nonsteroidal anti-inflammatory agents, finger splinting, and massage, with corticosteroid injections reserved for cases refractory to these conservative measures [5]. However, only 57% of patients experience resolution of recalcitrant trigger finger with corticosteroid injections [8]. Surgical release remains the mainstay for definitive treatment when conservative measures fail [1, 5, 9, 10]. Both open and percutaneous techniques have been described, with comparable functional outcomes and complication rates [9]. Ultrasound-guided percutaneous approaches have demonstrated greater success than procedures without sonographic guidance [11]. These less-invasive techniques do not require general anesthesia, can be performed in an outpatient setting, and lead to shorter recovery times, reduced pain at follow-up, and increased patient satisfaction when compared to open surgery [10].

Despite the advantages of ultrasound-guided techniques, the optimal choice of instrumentation remains unclear. Scalpel, knife-blade, tenotome, and hypodermic needle techniques have been described [11–13], but most studies have not reported specific outcomes. In this study, we therefore sought to evaluate patient outcomes with an ultrasound-guided percutaneous technique using small needles. Specifically, our goal was to assess the functional and symptomatic outcomes and safety of ultrasound-guided percutaneous TF release using standard small-gauge needle fenestration and to identify predictors of functional and symptomatic outcomes.

## Materials and methods

### Study population

This retrospective study was performed with approval by the Institutional Review Board with a waiver of informed consent. All ultrasound-guided A1 needle fenestration procedures completed between July 2020 and August 2024 were included in the study. All procedures were performed on an outpatient basis by one of two attending musculoskeletal radiologists with 13 and 7 years of experience at one of two locations with referral from an orthopedic or hand surgeon.

For each procedure, information on patient sex and age, fenestration site (laterality and digit), and procedure duration were collected from the medical records. In most cases, the clinical severity of finger triggering was classified immediately before and after intervention using the Quinnell grading system (grade 0, normal movement; grade I, uneven movement; grade II, actively correctable locking; grade III, passively correctable locking; and grade IV, fixed deformity) [14]. Classifications were assigned to affected digits based on physical examination findings during flexion and extension as described by Langer et al. [15].

### Procedure details

Patients were positioned supine with their arm and hand beside them, and the appropriate digit was marked along the volar aspect at the A1 pulley and sterilely prepared and draped. The finger was prepared and isolated so that a physical examination for triggering could be performed while the procedure was in progress. After appropriate safety checks were carried out, a “hockey-stick” small-footprint high-frequency linear array ultrasound transducer (Siemens Acuson S1000 14L5 [5–14 MHz] Hockey Stick transducer) was used to confirm the anatomical site, and 1% lidocaine was administered with a 25-gauge needle to achieve a digital block at the level of the A1 pulley.

**Fig. 1:**
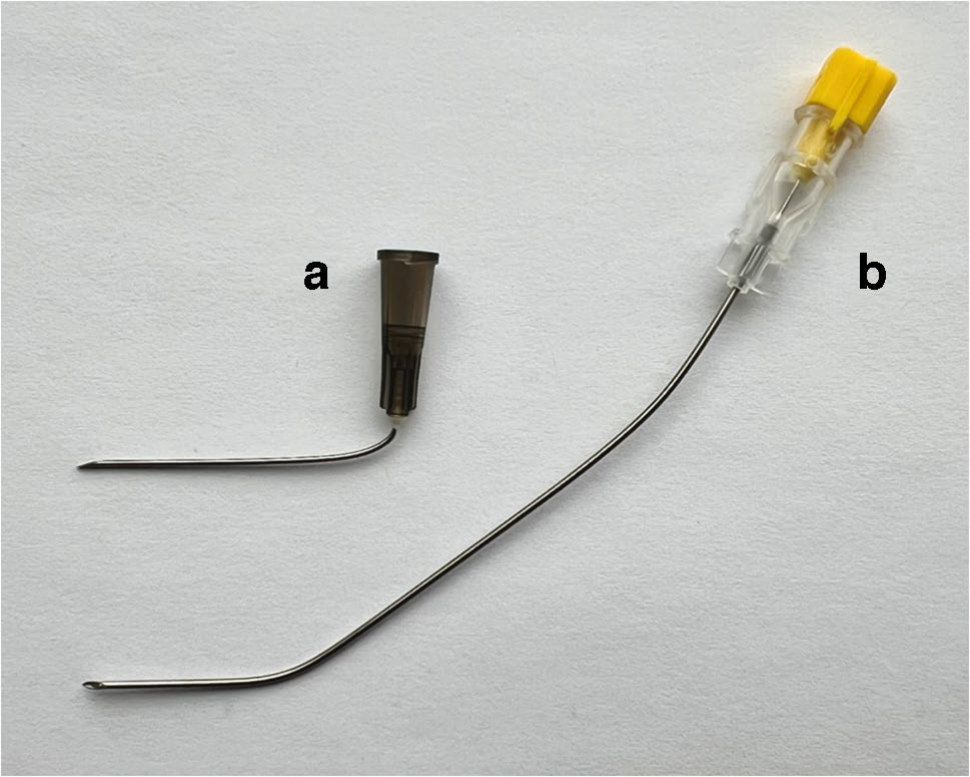
Needle preferences varied for the two operators. **a** One operator preferred a 1.5-inch 22-gauge hypodermic needle with a sharp bend near the base followed by a slight curve that flattens near the tip with an upward bevel. **b** The other operator preferred a 20-gauge spinal needle with a long curve over the entire needle and sharper short curve near the needle tip with bevel to the side. Both found 22-gauge needles to be suitable for most fenestrations

A 22-gauge needle was most commonly used for fenestration, with the needle manually bent based on the operator’s preference (Fig. 1). Typically, a 25-gauge needle was also bent and used to anesthetize the tendon sheath, and a 20-gauge needle was at times used to achieve a successful release. An ergonomic needle handle was developed by one of the operators to improve needle control and maintain fixed bevel orientation; this device was only used by the developer and only in more recent cases. Bending the needle was necessary to avoid injuring the tendon, as this allowed the operator to achieve a parallel course for the needle relative to the flexor tendon as the overlying A1 pulley was fenestrated. Bending of the needle was typically performed using one of two methods. Most commonly, a hard sterile surface was used to restrain the needle near the tip while the operator held the needle near the base and applied force toward the hard surface to achieve the desired bend. Alternatively, a larger gauge needle was cannulated with the fenestration needle, with the larger gauge needle used to bend the fenestration needle. Shorter hypodermic needles or longer spinal needles were used according to the operator preference.

**Fig. 2:**
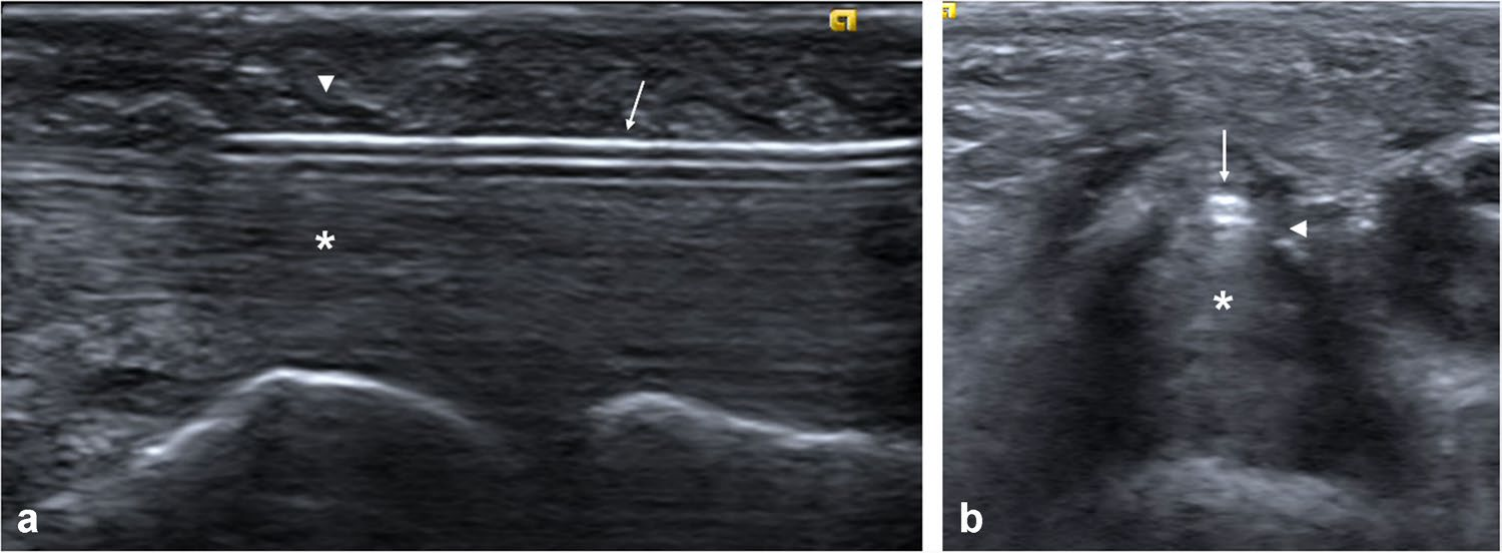
**a** The fenestration needle (arrow) is advanced to the A1 pulley (arrowhead) from distal to proximal and aligned superficial and parallel to the digit flexor tendons (asterisk (*)) under real-time sonographic guidance using a hockey-stick probe in a longitudinal plane. **b** Transverse imaging can also be used to ensure fenestration across the entire thickness of the A1 pulley

Once the fenestration needle was bent to the operator’s preference and the area and tendon sheath were sufficiently anesthetized, the fenestration needle was advanced to the A1 pulley from distal to proximal under real-time longitudinal sonographic guidance using a hockey-stick transducer (Fig. 2a). During fenestration, real-time transverse imaging was sometimes used to ensure fenestration across the entire thickness and appropriate central width of the A1 pulley. Transverse imaging was also used to ensure that the fenestration path was appropriately positioned within the medial–lateral aspect of the pulley and was not approaching either digital neurovascular bundle (Fig. 2b).

After a course was aligned through the A1 pulley parallel to the tendon, the A1 pulley was fenestrated by repeatedly traversing the pulley under real-time ultrasound guidance until a successful release was achieved. Upon initial fenestration, the pulley was found to have a tough, rubbery consistency. After numerous fenestrations, the needle was felt to glide smoothly just superficial to the tendon. At this point, both dynamic sonographic evaluation and physical examination were used to assess the result of the fenestration. Lifting the needle when positioned at the deeper aspect of the pulley was also used to assess A1 pulley laxity after release. Frequently, thickening and/or scarring of the tendon sheath occurred beyond the longitudinal extent of the A1 pulley, either proximally or distally. To the operator, this felt like small bands or threads of scars that could create a snapping sensation when fenestrated. These scarred or thickened areas could result in persistent triggering even when the pulley was felt to be released, so they were fenestrated, as well. Any small ganglia near the A1 pulley were also fenestrated when encountered.

Once a successful release was obtained, the area of the fenestrated/released A1 pulley was injected with 4 mg dexamethasone either through the same fenestration needle or through a 25-gauge needle. Patients were discharged after the procedure and were advised that they could experience soreness and possible bruising/swelling for up to 2 weeks. Over-the-counter pain medications were recommended as needed. Patients were also instructed to perform daily range-of-motion exercises for a month beginning the day after the procedure, typically advised simply to flex and extend their digit (typically by making a clenched fist then opening) several times in a row (~ 10) a few times a day (~ 3), in order to maintain the pulley release and prevent any scarring related re-stenosis of the tendon sheath at the pulley. This recommendation was emphasized as important even if the patient was experiencing some discomfort, persistent clicking, or uneven motion, and it otherwise was recommended to maintain any other interventions prescribed by the referring provider, including splinting.

### Baseline characteristics

Baseline information on patients was collected through a chart review, with information obtained from the latest office visit with the referring physician before the procedure. Data were collected regarding estimated symptom duration, pain at baseline (using the Visual Analog Scale [VAS]), and previous interventions used for symptom management (steroid injection or previous pulley fenestration/release).

### Follow-up review

For cases with follow-up data available, information was obtained via a chart review including data recorded through September 10, 2024. Functional status was reviewed at the first follow-up visit after the procedure and was categorized as worsened, unchanged, or improved based on subjective patient account and reported physical examination findings or clinical impression. The type and date of any additional interventions on the affected digit (steroid injection, repeat fenestration, surgical release) within the follow-up window were recorded for each case. If present, VAS pain scores reported at follow-up visits were recorded based on the time since the procedure (< 6 months, 6–12 months, or > 12 months). If multiple scores were reported within a time frame, the most recent score was recorded. Additionally, a final follow-up VAS pain score was recorded as the latest pain score reported across the entire follow-up period. For cases involving a repeat fenestration or surgical release, the latest score before the re-intervention was used. Lastly, for safety data, information about any complications including infection, tendon tear, or digital bowstringing reported within the study period was collected.

### Statistical analysis

The data were organized in Excel (Microsoft Corp., Redmond, WA, USA) and analyzed in GraphPad Prism (Version 10.5 of Prism 10 for Windows, Copyright © 2025 GraphPad Software, LLC) and SAS/STAT software (Version 9.4 of the SAS System for Windows, Copyright © 2019 SAS Institute Inc). Each data point in the analysis reflected a single fenestration, rather than an individual patient, as some patients underwent fenestrations on more than one digit. Descriptive statistics and total counts were calculated for the patient and procedure details discussed above. These were reported as mean ± standard deviation (SD) or median (interquartile range [IQR]), as appropriate. To test whether the fenestration procedure immediately affected triggering severity, the change in Quinnell grades from baseline to post-procedure were treated as ordinal variables in a multinomial model, adjusting for baseline Quinnell grade; Generalized Estimating Equations (GEEs) were used to account for the clustered nature of the data (i.e. multiple procedures in some patients). Potential predictors of the change in Quinnell grade from baseline to post-procedure were also investigated in the model including sex, procedure length, prior fenestration, and duration of symptoms. Similarly, a linear model was built for the change in pain scores from baseline, adjusting for baseline pain score and using GEEs to account for the clustered data.

Logistic regression with GEEs to account for the clustered data was performed to determine whether Quinell grade at procedure start, grade at procedure end, or difference in grade between start and end predicted the incidence of follow-up, need for additional intervention, or change in functional status at first follow-up. Similarly, the effect of these predictors on total pain reduction was assessed using a linear regression model with GEEs. To account for multiple comparisons, Holm’s method was used to adjust the p-values. Hypothesis testing for each model was performed using the Wald test of regression coefficient significance (α = 0.05), with odds ratios (ORs) and 95% confidence intervals (CIs) reported.

## Results

A total of 119 ultrasound-guided fenestrations were performed in 92 patients. Among these patients, 36 (39%) were men and 56 (61%) were women, with a mean patient age of 63 ± 13 years (range: 30–92 y) (Table 1). The median estimated duration of trigger finger symptoms before the procedure was 12 months (IQR: 7–18 mo). The median procedure length was 16 min from lidocaine administration to needle removal (IQR: 13–20 min). A 22-gauge needle was used most often (87 procedures, 73%), followed by 20-gauge (23 procedures, 19%) and 25-gauge (9 procedures, 8%) needles. The procedure was performed, from most to least often, on the long finger (46 digits, 39%), ring finger (39 digits, 33%), thumb (17 digits, 14%), index finger (14 digits, 12%), and little finger (3 digits, 3%).

**Table 1:** Patient and procedure characteristics (*n* = 119 fenestrations)

Characteristic	Value
Patient sex, *n* (%)	
Male	36 (39)
Female	56 (61)
Mean patient age ± SD, *y*	63 ± 13
Median symptom duration (IQR), mo	12 (7–18)
Median procedure length (IQR), min	16 (13–20)
Cases with previous intervention reported, *n* (%)	97 (82)
Steroid injection, *n* (%)	95 (80)
Fenestration, *n* (%)	5 (4)
Cases with follow-up intervention reported, *n* (%)	21 (18)
Steroid injection, *n* (%)	15 (13)
Repeat fenestration, *n* (%)	3 (3)
Surgical release, *n* (%)	5 (4)
Cases with Quinnell grade reported, *n* (%)	99 (83)
Median Quinnell grade at procedure start (IQR)	3 (2–4)
Median Quinnell grade at procedure end (IQR)	0 (0–1)
Cases with improved Quinnell grade, *n* (%)	99 (100)
Digits, *n* (%)	
Thumb	17 (14)
Index	14 (12)
Long	46 (39)
Ring	39 (33)
Little	3 (3)

Counts represent individual procedures, as several patients received interventions on multiple digits and are reported with percentages relative to total cases

*IQR* interquartile range, *SD* standard deviation

Most procedures were performed after previous interventions, with 95 (80%) following previous steroid injections and 5 (4%) representing re-fenestrations (Table 1). A few diabetic patients did not receive therapy with corticosteroid injections before or during the fenestration procedure because of concerns about poor glycemic control. Additional interventions on the same digit after fenestration were reported following 21 procedures (18%), with 15 (13%) subsequent steroid injections, 3 (3%) subsequent repeat fenestrations, and 5 (4%) subsequent surgical A1 pulley release procedures.

**Fig. 3:**
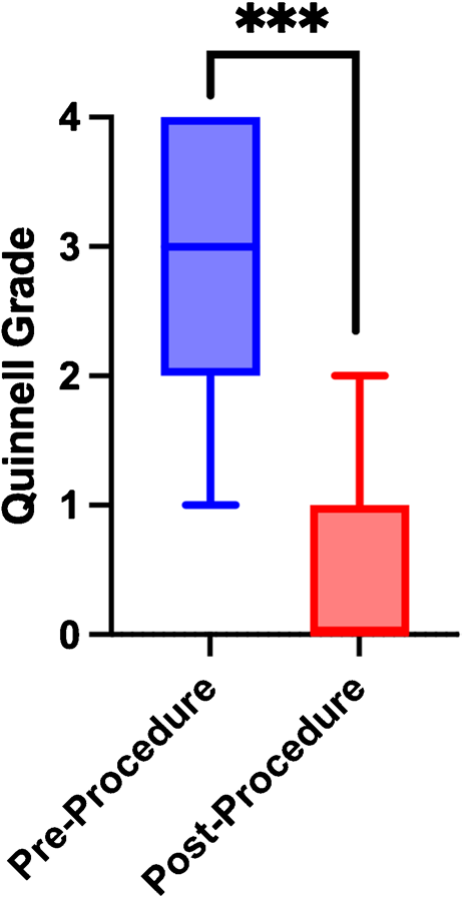
Box plots of Quinnell grades immediately before (blue) and after (red) the fenestration procedure displayed as median with IQR. Asterisks indicate *p* < 0.001

Periprocedural Quinnell grades were reported for 99 procedures (83%), with a median score at procedure start of 3 (IQR: 2–4) and a median score at procedure end of 0 (IQR: 0–1) (Table 1), which was a statistically significant reduction (*p* < 0.001) (Fig. 3). The specific Quinnell grades reported before and after the procedure are detailed in Table 2. Significant predictors of greater improvement in Quinnell grade were worse baseline Quinnell grade (*p* < 0.001), shorter duration of symptoms (*p* = 0.018), and no prior fenestration (*p* < 0.001).

**Table 2:** Change in Quinnell grade from procedure start to procedure end

Quinnell grade at procedure start	Quinnell grade at procedure end				
	0	I	II	III	IV
I	10	0	0	0	0
II	24	7	0	0	0
III	21	6	0	0	0
IV	19	10	2	0	0

Values shown are numbers of patients with proceduralist-determined Quinnell grades

Within the chart review window, follow-up information was recorded after 60 procedures (50%). Most follow-ups occurred as office visits (*n* = 56; 93%); the remaining follow-ups occurred as messages in the electronic medical record (*n* = 4; 7%). The median time from procedure date to first follow-up was 34 days (IQR: 27–52 d). Of the 60 procedures for which follow-up data was available, 42 (70%) demonstrated functional improvement of digit triggering at the first follow-up; the remaining 18 cases (30%) had experienced no change in function. There were no reported cases of worsened function at the first follow-up visit. There were also no reports of infection, tendon tear, or digital bowstringing.

**Fig. 4:**
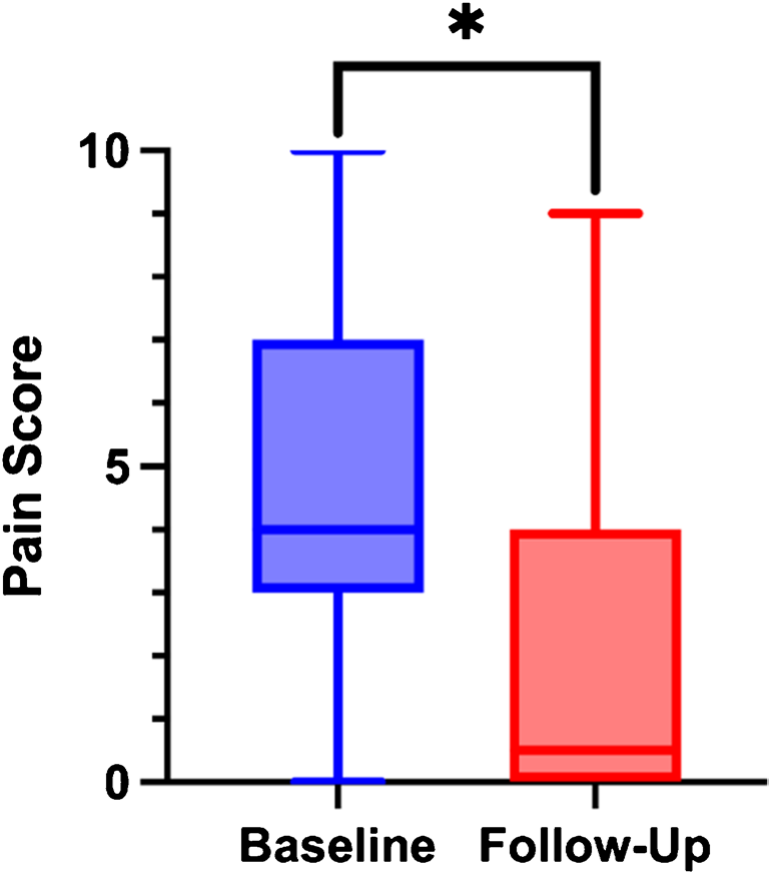
Box plots of pain scores reported at baseline and last available follow-up scores displayed as median with IQR. Pairwise comparisons were performed using the Wilcoxon matched-pairs signed-rank test. Asterisks indicate *p* = 0.046

Baseline and follow up pain scores were available for 46 procedures (39%), with a median reported baseline score of 4 (IQR: 3–7) and 0.5 (IQR: 0–4) at last available follow-up (Table 3). Pain scores at last available follow-up were significantly decreased since baseline (*p* = 0.046) (Fig. 4).

**Table 3:** VAS pain score progression over follow-up period

Variable	Median value (IQR) [# procedures]
Pain score at baseline	4 (3–7) [110]
Change in pain score	
< 6 months	− 2.50 (− 4.00 to 0) [49]
6–12 months	− 3.00 (− 5.5 to − 0.75) [15]
> 12 months	− 4.50 (− 6.75 to − 1.5) [7]

*IQR* interquartile range, *VAS* Visual Analog Scale

Smaller improvement in Quinnell grade from baseline to follow-up was a significant predictor of the likelihood of an additional intervention (OR = 0.48, 95% CI: 0.33, 0.70; adjusted *p* < 0.001). Follow-up was more likely when the end Quinnell grade was higher (OR = 3.19, 95% CI: 1.45, 7.01; adjusted *p* = 0.012), while improved functional status tended to be more likely when the end Quinnell grade was lower (OR = 0.25, 95% CI: 0.08, 0.79; adjusted *p* = 0.054).

## Discussion

In this study, we demonstrated the efficacy and safety of an ultrasound-guided A1 pulley fenestration release technique using standard small-gauge needles as a treatment for TF in an outpatient setting. The procedure resulted in a functional release of the A1 pulley, which was observable directly at the time of the procedure via examination and dynamic ultrasound assessment. Among the patients with retrospective follow-up available (approximately half of the study patients), 70% reported functional improvement. However, the true percentage with functional improvement was likely higher, as patients without a reason to follow up presumably were improved, and functional improvement was more commonly directly observed at the conclusion of the procedure. This is corroborated by the finding that higher end-procedure Quinnell grades were a significant predictor of follow-up occurrence. There were no complications of postprocedural infection, tendon tear, or bowstringing reported in the procedure reports or in the follow-up visits.

For patients with a locked digit or a digit that strongly triggered every time, the functional results were immediately apparent to the patient and operator at the conclusion of the procedure. In fact, the operator would typically continue the procedure until a successful functional outcome was achieved. For patients with milder or waxing and waning symptoms that were milder at the time of the procedure, the dynamic sonographic evaluation was helpful in assessing for sufficient release.

The most common intervention needed after A1 pulley fenestration release in this study was an additional steroid injection (13%). Typically, this was needed in patients who had pain within the first few weeks after the procedure that could not be controlled with over-the-counter medications. These patients would often follow up with the referring provider, who would clinically identify a postprocedural tenosynovitis and offer a steroid injection in the clinic. Most of these patients had functional improvement in their triggering symptoms after the postprocedural tenosynovitis resolved. It is not clear why some patients developed a postprocedural tenosynovitis despite the administration of a steroid injection at the conclusion of the fenestration procedure, but for affected patients, a repeat injection was sufficient to resolve the postprocedural pain.

Some patients did require repeat fenestration or a surgical release procedure. Often, these were challenging cases in which the release appeared to necessitate fenestration of thickened tendon sheath fibers beyond the confines of the A1 pulley. These more challenging cases often demonstrated persistent clicking or uneven motion despite thorough fenestration with a 22-gauge needle. Both operators found that in some of these cases, a 20-gauge needle provided more effective fiber disruption, likely because of the increased stiffness and size of the needle. Although not captured in the data window for this study, 18-gauge needles have been very rarely used for difficult or repeat pulley release. Most cases, however, were able to be effectively released with a 22-gauge needle, and some less severe cases were able to be released with 23- or 25-gauge needles.

Although both operators found 22-gauge needles to be suitable for most fenestration procedures, they had slightly different preferences regarding the needle length and curvature. One operator preferred a 1.5-inch 22-gauge hypodermic needle with a sharp bend near the base followed by a slight curve that flattened near the tip. The other operator preferred a 20-gauge spinal needle with a long curve over the entire needle and sharper short curve near the needle tip. This operator performed fenestrations with the stylet in the spinal needle to simulate a cutting tip.

As the operators became more accustomed to the procedure, it became clear to referring providers and the operators that patients with recurrent or nonresolving triggering did not necessarily have to undergo surgery but could instead be treated with repeat fenestration procedures to achieve successful triggering relief. Note that some patients, despite improvement, did demonstrate ongoing mechanical symptoms. Some of these cases did anecdotally resolve over time with consistent range-of-motion exercises (flexion extension). A few, however, did subsequently undergo surgery (5 of 119, 4.2%), repeat fenestration (3 of 119, 2.5%), or steroid injection (15 of 119, 13%) procedures. Previous research examining complication rates with surgical release yielded similar results, with Everding et al. [16] demonstrating an overall complication rate for surgical trigger finger release of 12% (citing a range of 1%−43% in the literature) and a reoperation rate of 2.4% (citing a range of 0–3.8% in the literature).

This study had several limitations. The inclusion of a control group of patients who received injections only without fenestration release would have been optimal to explicitly demonstrate the comparative benefit of fenestration release with injections versus injections only; however, most procedures in this study (80%) occurred in patients who already received steroid injections and were undergoing the procedure because of refractory symptoms. The evolution of operator experience and technique over time, as well as differences in operator preferences, resulted in some variability in procedure technique, and the study was not powered to detect small differences in outcomes from these minor variations. However, the good outcomes we observed despite these differences do support the generalizability of this technique, suggesting that clinicians can adapt the procedure based on their own preferences. Similarly, as additional patients were referred for fenestration at an increased rate over time, the severity of cases tended to decrease at an earlier phase of disease and with less prior interventions, which may also have affected the outcomes. In this study, Quinnell grading was a primary outcome measure of functional improvement after fenestration. Although the clinical exam based Quinnell system is widely used clinically and academically to assess trigger finger severity, published data on its inter- and intra-rater reliability are limited and was not assessed in this study between the two experienced operators using the standardized criteria. This retrospective study had follow-up clinical information for only 50% of cases, with statistically significant bias toward less favorable outcomes for those who did have follow-up data available. Finally, not enough cases were available to sufficiently assess for outcomes differences with differences in fenestration techniques.

In conclusion, we found that percutaneous ultrasound-guided A1 pulley fenestration release can be effectively performed as an outpatient office procedure and can serve as an alternative to TF release surgery in the operating room. At our institution, surgeons and other clinical colleagues who know about the procedure often refer patients for fenestration, with surgeons tending to refer cases that may be less severe, as well as patients with a higher surgical risk profile. Cost-effectiveness studies are needed to determine whether fenestration is appropriate only in the setting of steroid-recalcitrant TF or if fenestration performed earlier in the disease process could lead to improved outcomes, obviating the need for additional procedures.

## Data Availability

The data that support the findings of this study are available for scientific purposes from the corresponding author upon reasonable request subject to IRB approval and removal of personal health information.

## References

[1] Makkouk AH, Oetgen ME, Swigart CR, Dodds SD. Trigger finger: etiology, evaluation, and treatment. Curr Rev Musculoskelet Med. 2008;1(2):92–6. 19468879 10.1007/s12178-007-9012-1 PMC2684207

[2] Koh S, Nakamura S, Hattori T, Hirata H. Trigger digits in diabetes: their incidence and characteristics. J Hand Surg Eur. 2010;35(4):302–5. 10.1177/1753193409341103 19687073

[3] Weilby A. Trigger finger. Incidence in children and adults and the possibility of a predisposition in certain age groups. Acta Orthop Scand. 1970;41(4):419–27. 5502406 10.3109/17453677008991529

[4] Kumar P, Chakrabarti I. Idiopathic carpal tunnel syndrome and trigger finger: is there an association? J Hand Surg Eur. 2009;34(1):58–9. 10.1177/1753193408096015 18936127

[5] Matthews A, Smith K, Read L, Nicholas J, Schmidt E. Trigger finger: an overview of the treatment options. JAAPA. 2019;32(1):17–21. 30589729 10.1097/01.JAA.0000550281.42592.97

[6] Chuang XL, Ooi CC, Chin ST, et al. What triggers in trigger finger? The flexor tendons at the flexor digitorum superficialis bifurcation. J Plast Reconstr Aesthet Surg. 2017;70(10):1411–9. 28709917 10.1016/j.bjps.2017.05.037

[7] Bianchi S, Gitto S, Draghi F. Ultrasound features of trigger finger: review of the literature. J Ultrasound Med. 2019;38(12):3141–54. 31106876 10.1002/jum.15025

[8] Fleisch SB, Spindler KP, Lee DH. Corticosteroid injections in the treatment of trigger finger: a level i and ii systematic review. J Am Acad Orthop Surg. 2007;15(3):166–71. 17341673 10.5435/00124635-200703000-00006

[9] Wang J, Zhao JG, Liang CC. Percutaneous release, open surgery, or corticosteroid injection, which is the best treatment method for trigger digits? Clin Orthop Relat Res. 2013;471(6):1879–86. 23208122 10.1007/s11999-012-2716-6 PMC3706641

[10] Yavari M, Modaresi SM, Hassanpour SE, Moosavizadeh SM, Tabrizi A. Clinical study between percutaneous ultrasound-guided release and open classic surgery in treating multiple trigger fingers. Adv Biomed Res. 2023;12:88. 37288036 10.4103/abr.abr_392_21 PMC10241637

[11] Zhao JG, Kan SL, Zhao L, et al. Percutaneous first annular pulley release for trigger digits: a systematic review and meta-analysis of current evidence. J Hand Surg Am. 2014;39(11):2192–202. 25227600 10.1016/j.jhsa.2014.07.044

[12] Singh DK, Chari B. Ultrasound-guided percutaneous release of pulley in trigger finger: a curved needle technique. J Arthroscopy Joint Surg. 2023;10(3):125.

[13] Rajeswaran G, Lee JC, Eckersley R, Katsarma E, Healy JC. Ultrasound-guided percutaneous release of the annular pulley in trigger digit. Eur Radiol. 2009;19(9):2232–7. 19399506 10.1007/s00330-009-1397-3

[14] Quinnell RC. Conservative management of trigger finger. Practitioner. 1980;224(1340):187–90. 7367373

[15] Langer D, Maeir A, Michailevich M, Luria S. Evaluating hand function in clients with trigger finger. Occup Ther Int. 2017;2017:9539206. 29097982 10.1155/2017/9539206 PMC5612741

[16] Everding NG, Bishop GB. Risk factors for complications of open trigger finger release. Hand. 2015;10(2):297–300. 26034447 10.1007/s11552-014-9716-9 PMC4447687

